# Shear-Wave Elastography in Musculoskeletal Pain: Current Evidence and Clinical Applications for Tissue Stiffness Assessment

**DOI:** 10.3390/medicina62102011

**Published:** 2026-10-18

**Authors:** Hortensia De la Corte-Rodríguez, Beatriz A. Rodríguez-Damiani, Natalia Pinilla-Téllez, Emérito-Carlos Rodríguez-Merchán, Juan Manuel Román-Belmonte

**Affiliations:** 1 Department of Physical Medicine and Rehabilitation, Hospital Universitario La Paz, 28046 Madrid, Spain; hortensiadelacorterodriguez@gmail.com (H.D.l.C.-R.); nataliapinillat@gmail.com (N.P.-T.); 2 IdiPaz Institute for Health Research, 28046 Madrid, Spain; 3 Facultad de Medicina, Alfonso X El Sabio University, 28691 Madrid, Spain; bearoddamiani@gmail.com (B.A.R.-D.); juanmaromanbelmonte@gmail.com (J.M.R.-B.); 4 Department of Physical Medicine and Rehabilitation, Hospital Central de la Cruz Roja San José and Santa Adela, 28003 Madrid, Spain; 5 Department of Orthopedic Surgery, Hospital Universitario La Paz, 28046 Madrid, Spain

**Keywords:** shear-wave elastography, musculoskeletal pain, plantar fasciitis, Achilles tendinopathy, patellar tendinopathy, carpal tunnel syndrome, spinal pain, myofascial pain

## Abstract

*Background and Objectives*: Shear-wave elastography (SWE) is a quantitative ultrasound technique that estimates tissue stiffness based on the propagation velocity of shear waves. In musculoskeletal pain, it can provide biomechanical information that complements clinical examination and conventional ultrasound, although its relationship with pain has not been fully established. *Materials and Methods*: A narrative review was conducted of the literature indexed in PubMed, Embase, Web of Science, Cochrane Library, CINAHL, and PEDro on SWE and musculoskeletal pain. Priority was given to studies with the strongest evidence and the greatest clinical relevance. *Results*: SWE allows for the assessment of muscles, tendons, fascia and peripheral nerves, but its results depend on technical and biological factors, such as position, tension, muscle contraction, probe orientation and the equipment used. The evidence is more consistent in conditions with known tissue alterations, such as plantar fasciitis, Achilles or patellar tendinopathy and carpal tunnel syndrome, where it can support diagnosis and may contribute to clinical assessment and treatment monitoring. In other conditions, such as spinal pain and myofascial pain, where the structure causing the clinical symptoms is not so well defined, SWE findings are more heterogeneous. Currently, there are no fully accepted ultrasound parameters of clinical relevance, and comparisons between studies remain limited by methodological variability. *Conclusions*: SWE is a useful complementary tool in musculoskeletal pain, but not yet a standalone diagnostic modality or an objective measure of pain. Its clinical role will depend on standardized protocols, validated reference values, and higher-quality studies assessing its diagnostic, prognostic, and treatment-monitoring value.

## 1. Introduction

Musculoskeletal pain is a complex clinical phenomenon in which nociceptive, neuropathic, and nociplastic mechanisms may coexist, while inflammation and mechanical alterations may contribute to the generation and persistence of nociceptive activity [1]. In clinical practice, patients frequently report stiffness, tension, or localized pain that does not always correlate with findings on conventional imaging techniques. Conversely, structural abnormalities are also frequently observed in asymptomatic individuals [2]. This discrepancy between symptoms and morphological findings has increased interest in techniques such as shear-wave elastography (SWE), which enable non-invasive characterization of the mechanical properties of tissues in vivo [3].

From a mechanistic perspective, nociceptive musculoskeletal pain is associated with activation of peripheral nociceptors in response to actual or threatened tissue injury and may be influenced by mechanical loading, local inflammatory processes, and degenerative tissue changes. Neuropathic pain results from a lesion or disease affecting the somatosensory nervous system and may occur, for example, in peripheral neuropathies or nerve-entrapment disorders. Nociplastic pain reflects altered nociceptive processing when neither ongoing peripheral tissue damage nor a lesion of the somatosensory system is sufficient to explain the clinical presentation. These mechanisms are not mutually exclusive and may coexist, particularly in chronic musculoskeletal pain [1]. Tissue injury, repetitive mechanical loading, inflammation, edema, collagen disorganization, degeneration, fibrosis, and changes in muscle activation or neural mechanical properties may alter the biomechanical behavior of muscles, tendons, fasciae, ligaments, and peripheral nerves. Depending on the tissue and pathological process involved, these alterations may result in either increased or decreased stiffness. However, changes in mechanical properties represent only one component of the multidimensional mechanisms underlying musculoskeletal pain and should not be assumed to have a direct or causal relationship with symptom intensity [1,2].

Several imaging-based techniques can be used to characterize tissue structure and mechanical properties. Conventional B-mode ultrasonography provides high-resolution information regarding morphology, echogenicity, thickness, architecture, and dynamic behavior, but does not directly quantify tissue stiffness. Strain elastography estimates relative tissue deformation in response to externally or physiologically applied compression and provides predominantly qualitative or semiquantitative information; however, its measurements can be influenced by the magnitude and uniformity of the applied compression and therefore remain relatively operator-dependent. Transient elastography generates mechanically induced shear waves and estimates tissue stiffness from their propagation velocity, although its application has predominantly involved relatively homogeneous tissues and may be more difficult in small, superficial, or strongly anisotropic musculoskeletal structures. Magnetic resonance elastography similarly evaluates the propagation of mechanically induced waves and can provide quantitative and potentially volumetric assessment of deep tissues, but requires specialized equipment, is more expensive, and is less widely available than ultrasound-based approaches [4].

SWE is one of the most extensively investigated ultrasound elastography modalities in the musculoskeletal field. Unlike conventional B-mode ultrasonography, which primarily provides information on morphology and echotexture, SWE enables quantitative estimation of tissue mechanical properties. The technique generates shear waves using acoustic impulses and measures their propagation velocity, which increases in stiffer tissues. Results can be expressed either as shear-wave velocity in meters per second or as an estimated elastic modulus in kilopascals [4,5].

This technique is rapidly evolving, with new applications and potential clinical uses emerging in the field of musculoskeletal imaging. By enabling the assessment of tissue mechanical properties and elasticity, SWE complements the diagnostic information provided by grayscale or B-mode ultrasonography, color Doppler, and power Doppler imaging [6].

Importantly, SWE does not measure pain itself. It quantifies tissue mechanical behavior, typically through shear-wave propagation velocity or derived elastic parameters, and these measurements should therefore be interpreted as biomechanical biomarkers rather than direct indicators of pain intensity. Mechanical abnormalities may be present in individuals without symptoms, whereas patients with clinically relevant pain may show little or no measurable alteration in tissue stiffness; consequently, the relationship between SWE parameters and pain or disability is condition- and tissue-dependent [2]. SWE is the focus of the present review because it combines quantitative assessment of tissue mechanical properties with real-time ultrasonography, allowing mechanical information to be obtained alongside conventional anatomical imaging during the same examination. Its relatively high spatial resolution, dynamic capability, absence of ionizing radiation, and integration into increasingly available ultrasound platforms make it particularly attractive for the assessment of superficial musculoskeletal tissues and for repeated or longitudinal measurements. Nevertheless, these advantages must be balanced against important technical limitations, including tissue anisotropy and sensitivity to probe orientation, applied pressure, acquisition depth, patient positioning, and equipment-specific algorithms [4,5,7].

SWE is available on multiple ultrasound platforms, although its implementation in routine musculoskeletal clinical practice remains variable [7]. The aim of this article is to analyze the current role of SWE in musculoskeletal pain.

## 2. Methods

A targeted literature search was conducted between 1 July and 15 July 2026, using PubMed, the Cochrane Database, EMBASE, Web of Science, CINAHL, and PEDro. The search terms included “shear-wave elastography”, “musculoskeletal pain”, “spine”, “muscle”, “tendon”, “fascia”, and “nerve”. The final search covered publications available up to 15 July 2026. The review was restricted to articles published in English or Spanish. This narrative review was intended to provide a selective synthesis of clinically relevant evidence regarding the applications and limitations of SWE in musculoskeletal pain, rather than an exhaustive systematic review of all available publications.

Publications were considered according to their relevance to the clinical applications addressed in this review, their methodological characteristics, and their contribution to the existing evidence. When studies with different levels of evidence were available for the same clinical indication, systematic reviews, meta-analyses, and randomized clinical trials were preferentially considered. When higher-level evidence was limited or unavailable, observational studies and other lower-level evidence were included to provide a broader understanding of the available findings.

Particular consideration was given to studies published within the preceding five years that provided additional information not adequately covered by earlier publications, studies addressing less frequently investigated applications of SWE, such as peripheral neuropathies, and publications describing SWE acquisition protocols relevant to methodological standardization. Studies addressing SWE exclusively in non-musculoskeletal conditions, including liver, breast, thyroid, or prostate disorders, were considered outside the scope of this review.

The selection of publications was guided by their scientific and clinical relevance and the level of available evidence. Accordingly, the literature selection is presented as a qualitative narrative synthesis rather than as a systematic screening and eligibility process.

## 3. Physical Principles of SWE

A shear wave is a type of transverse wave that propagates through an elastic medium when the medium is repeatedly subjected to shear forces. Shear refers to a deformation, caused by two equal and opposite forces applied parallel to a material layer’s opposing surfaces, that changes its shape without altering its volume. Following deformation, the initial tissue layer returns to its original configuration, while adjacent layers undergo successive deformation. This process transmits the disturbance from one layer to another, allowing the wave to propagate transversely through the tissue [8].

Shear waves propagate through soft tissues at a velocity approximately one thousand times lower than that of conventional longitudinal ultrasound waves and attenuate approximately ten thousand times faster. Their propagation can be visualized, and their velocity quantified, using ultrasonography, provided that the system is capable of acquiring echoes at a sufficiently high frame rate [9].

**Figure 1:**
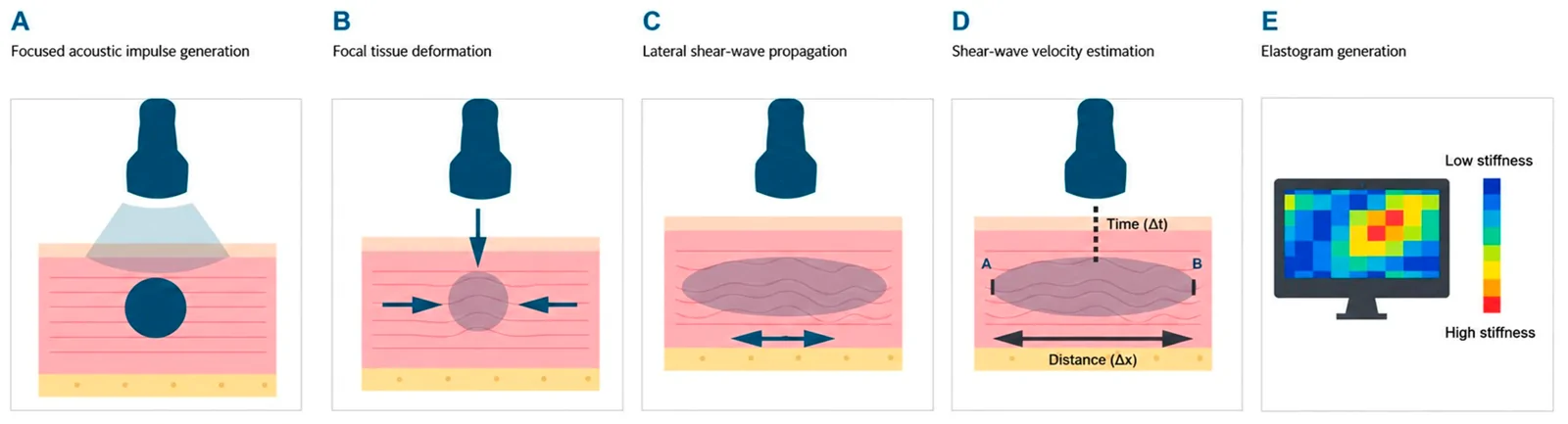
Generation and acquisition mechanism of shear-wave elastography (SWE). (**A**) The transducer emits a focused acoustic impulse (acoustic radiation force) into the musculoskeletal tissue. (**B**) This produces a focal tissue deformation through paired, opposing shear forces, changing the tissue’s shape without altering its volume [8]. (**C**) A shear wave propagates laterally through the adjacent tissue planes, at a velocity far lower than that of conventional ultrasound [9]. (**D**) Tissue displacement is tracked using ultrafast acquisition sequences, and the shear-wave velocity (cs) is derived from the time (At) needed to travel a known distance (Ax) between two points, A and B: c = Ax/At [10]. (**E**) Velocity values are ”color-coded” into an elastogram, “from low (blue) to high (red) stiffness,” and expressed as wave velocity (m/s) or estimated elastic modulus (kPa) [10].

On the ultrasound system display, the spatial distribution of tissue stiffness is represented by a color-coded elastogram. Quantitative results can be expressed either as shear-wave propagation velocity in meters per second or as an estimate of the elastic modulus in kilopascals [5]. The mechanism of SWE is shown in Figure 1.

## 4. Physical Limitations of Swe

Estimating shear-wave velocity requires the propagation of the wave to be recorded with high temporal resolution. For this reason, elastography systems use ultrafast acquisition sequences, generally achieving several thousand frames per second. An adequate tracking frame rate allows more accurate detection of the small tissue displacements induced by the shear wave and improves the stability of elastographic measurements [10].

The quality indicator is derived from parameters related to the intensity, regularity, and reliability of the detected wave propagation, although the specific algorithm varies between manufacturers. When the ultrasound signal is weak, tissue motion cannot be tracked reliably, or shear-wave propagation is insufficient, confidence in the resulting estimate decreases. Similar difficulties arise in fluid media, in which a valid elastographic measurement generally cannot be obtained [11].

One of the main limitations of SWE is its limited ability to assess structures at greater depths. When very superficial structures are examined, a layer of ultrasound gel approximately 5 mm thick may be placed between the skin and the transducer to improve image acquisition [12].

Quantification may also be influenced by the size, shape, and location of the region of interest, the adjustment options for which differ among ultrasound systems. The time required to acquire and stabilize the elastogram and the sensitivity of the technique to motion may limit the assessment of structures that change position rapidly. In addition, transducer pressure, insonation angle, and probe orientation relative to the anatomical fiber direction may alter the measured shear-wave velocities and calculated elastic moduli. Therefore, transducer position, image orientation, acquisition depth, region of interest (ROI), and applied pressure should be standardized [13,14,15].

## 5. Swe in Musculoskeletal Pain

In general, shear waves tend to propagate at higher velocities in healthy tendons than in tendons affected by tendinopathy and more rapidly when tendons and muscles are under tension or contracting than when they are relaxed. Furthermore, in healthy tendons, shear-wave velocity is generally higher in the longitudinal than in the transverse direction [16].

Because muscles become stiffer during contraction, SWE has recently been proposed as a potentially useful method for estimating individual muscle function, as muscle stiffness may serve as an indirect measure of muscle activation and force [17].

In patients with neuropathies, nerves show higher shear-wave propagation velocities than healthy nerves. In addition, the elastic properties of certain nerves can be assessed non-invasively while the related anatomical structures are passively mobilized [18].

Normal ligaments generally exhibit intermediate shear-wave propagation velocities at rest, which increase when the ligaments are placed under tension [19]. SWE has also been used to assess pain of rheumatological origin. Furthermore, shear-wave velocity in gouty tophi has been reported to vary according to their consistency, with higher values in harder tophi than in softer tophi or thickened synovium [8].

Importantly, the direction of SWE abnormalities is tissue- and disease-specific. Increased stiffness should not be interpreted as a universal marker of musculoskeletal pain or greater disease severity. Some painful muscular conditions and peripheral neuropathies are associated with increased SWE-derived stiffness, whereas plantar fasciopathy and several tendinopathies are more commonly associated with reduced shear-wave velocity or stiffness. Consequently, SWE measurements must be interpreted in relation to the specific tissue, pathological process, loading or contraction state, anatomical orientation, and acquisition protocol. SWE quantifies mechanical tissue behavior rather than pain itself.

SWE may also be useful for characterizing benign and malignant musculoskeletal soft-tissue tumors and for evaluating their changes during treatment. Some authors have found no significant quantitative or qualitative association between shear-wave velocity and lesion malignancy [20]. Other studies, however, have reported that longitudinal shear-wave velocity was approximately 30% lower in malignant than in benign masses. In addition, measurements obtained along the longitudinal and transverse axes showed a moderate correlation with each other [21]. Therefore, the role of SWE in assessing the malignancy of musculoskeletal tumors has not yet been clearly established.

The main applications of SWE in the different disorders associated with musculoskeletal pain are described below and summarized in Table 1.

## 6. Mechanical Neck Pain

Neck pain is a common health problem and is defined as pain located in the region extending from the superior nuchal line to the first thoracic vertebra and, laterally, to the insertions of the trapezius muscle near the shoulder joint. In most patients, its precise origin cannot be determined; therefore, it is generally considered a disorder of nonspecific etiology [55]. Neck pain ranks fourth among the leading causes of years lived with disability. Its estimated prevalence is approximately 20%, and up to 70% of the population may experience neck pain at some point during their lifetime. In addition, neck pain has a high tendency to recur [56].

SWE has been used to assess the elastic properties of muscles commonly associated with chronic neck pain. In a study of 42 patients with mechanical neck pain, 168 ultrasound images were acquired at the C7 vertebral level to evaluate anterior scalene muscle stiffness. The results showed higher stiffness values than those previously reported elasticity parameters measured using SWE for asymptomatic individuals in other studies [22].

Another study evaluated the ability of different parameters obtained using B-mode ultrasonography and SWE to distinguish patients with chronic nonspecific neck pain from asymptomatic individuals. No significant between-group differences were found for most of the variables analyzed, except for fatty infiltration, which was greater in patients with chronic neck pain. However, because this finding showed only moderate diagnostic accuracy and the remaining parameters had limited discriminatory ability, the authors recommended that SWE should not be used as a standalone technique to identify patients with idiopathic neck pain [23].

In addition to the anterior scalene muscle, SWE has been used to investigate the role of other muscles in neck pain. A study involving 109 participants, including 51 patients with chronic pain in the right posterior cervical region and 58 asymptomatic individuals, identified alterations in the elastic properties of the trapezius muscle. Patients with moderate-to-severe neck pain showed higher muscle stiffness values [24].

Postural abnormalities as a potential contributor to neck pain have also been investigated using SWE. A study involving 39 women assessed increased cervical muscle stiffness in patients with neck pain associated with postural abnormalities and reported a positive correlation between stiffness of the left upper trapezius muscle and shoulder angle, with muscle stiffness increasing as the shoulder angle increased [25].

In addition to pain, SWE has been used to assess related outcomes such as disability. One study compared upper trapezius stiffness using SWE in office workers with and without chronic neck pain. A total of 88 workers were included, 66 with chronic neck pain and 22 healthy controls. Patients with neck pain showed significantly greater upper trapezius stiffness, which was also associated with greater neck-related disability, stronger fear-avoidance beliefs, and lower work ability [26].

## 7. Mechanical Low Back Pain

Low back pain is characterized by pain located between the lower costal margin and the gluteal folds and may or may not be accompanied by pain radiating into the lower extremities. It is estimated to affect approximately 619 million people worldwide and is the leading global cause of years lived with disability. Its prevalence progressively increases with age and peaks at approximately 85 years. While acute episodes of nonspecific low back pain often resolve spontaneously, chronic forms generally have a less favorable course [57].

The quadratus lumborum muscle plays an important role in patients with low back pain. One study assessed the intra- and inter-rater reliability of SWE for quantifying quadratus lumborum stiffness in patients with low back pain. The technique showed excellent intra-rater reproducibility, indicating a high degree of consistency when measurements were performed by the same examiner [27].

A cross-sectional study used SWE to compare the stiffness of the thoracolumbar fascia, erector spinae muscles, and multifidus muscle in 30 patients with chronic nonspecific low back pain and 32 healthy individuals. The possible relationships between these mechanical properties and pain intensity and disability were also investigated. Overall, patients with low back pain showed greater stiffness of the lumbar muscular and fascial structures, although the magnitude of the differences varied according to the region examined and was more pronounced at the L4–L5 levels. Increased stiffness, particularly in the thoracolumbar fascia, may also be associated with greater pain intensity and poorer functional status [28].

The psoas muscle has also been investigated using SWE because of its potential role in mechanical low back pain. One study examined the relationship between psoas major muscle characteristics, particularly stiffness, and the presence of chronic low back pain in elite athletes. The study included 99 athletes from gymnastics and wushu teams, 53 of whom had chronic low back pain. The results showed that psoas major stiffness, unlike muscle strength or flexibility, was associated with low back pain and greater functional impairment, particularly when pain affected the dominant side [29].

SWE has also been used to classify mechanical low back pain as acute, subacute, or chronic. A study involving 52 patients with nonspecific low back pain found that combining B-mode ultrasound features with SWE parameters improved classification performance. Furthermore, elasticity parameters measured using SWE were identified among the most relevant factors for differentiating these patients [30].

Another potential application of SWE is the prediction of the risk of developing mechanical low back pain. A study involving 197 pregnant women developed a model to predict the risk of low back pain during the early postpartum period based on SWE assessment of the rectus abdominis muscle [31].

## 8. Myofascial Pain

**Figure 2:**
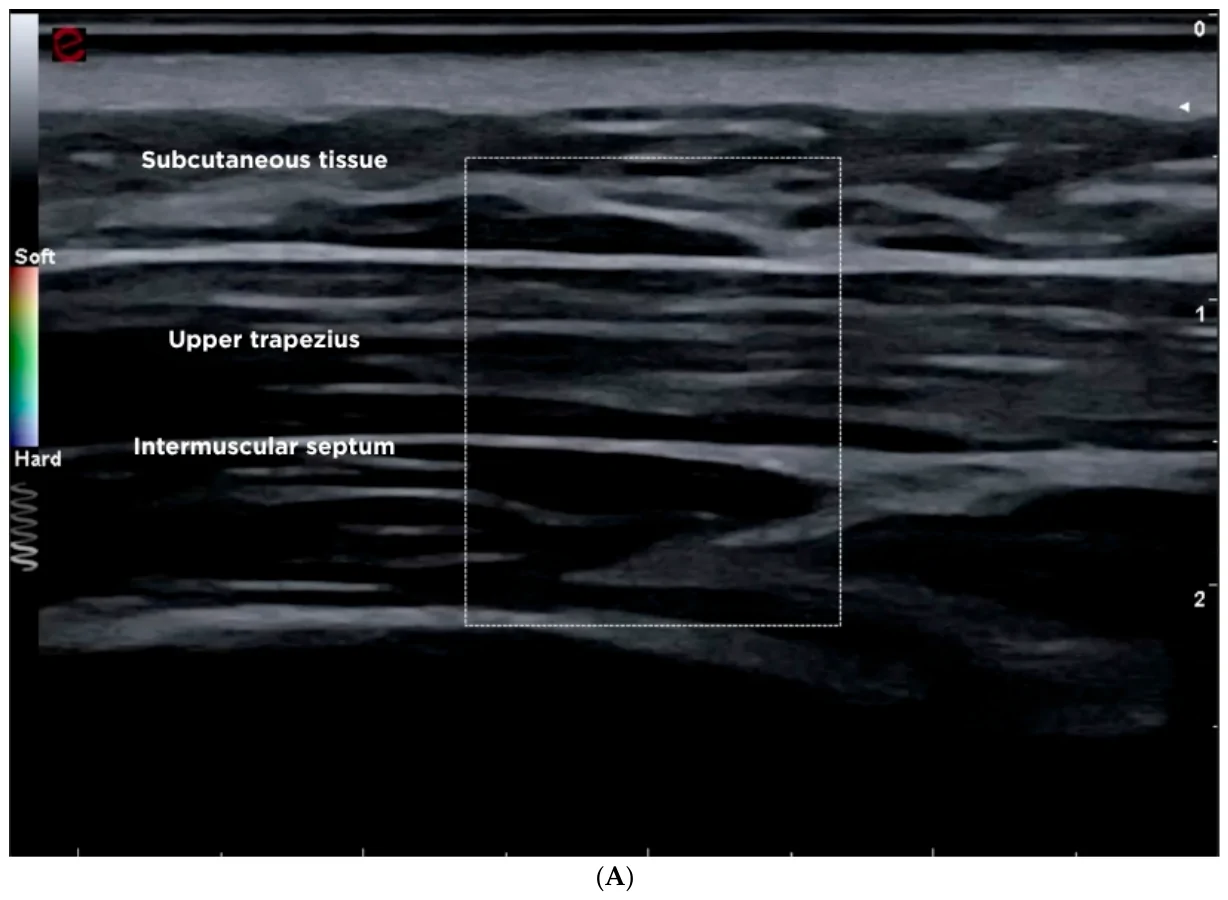

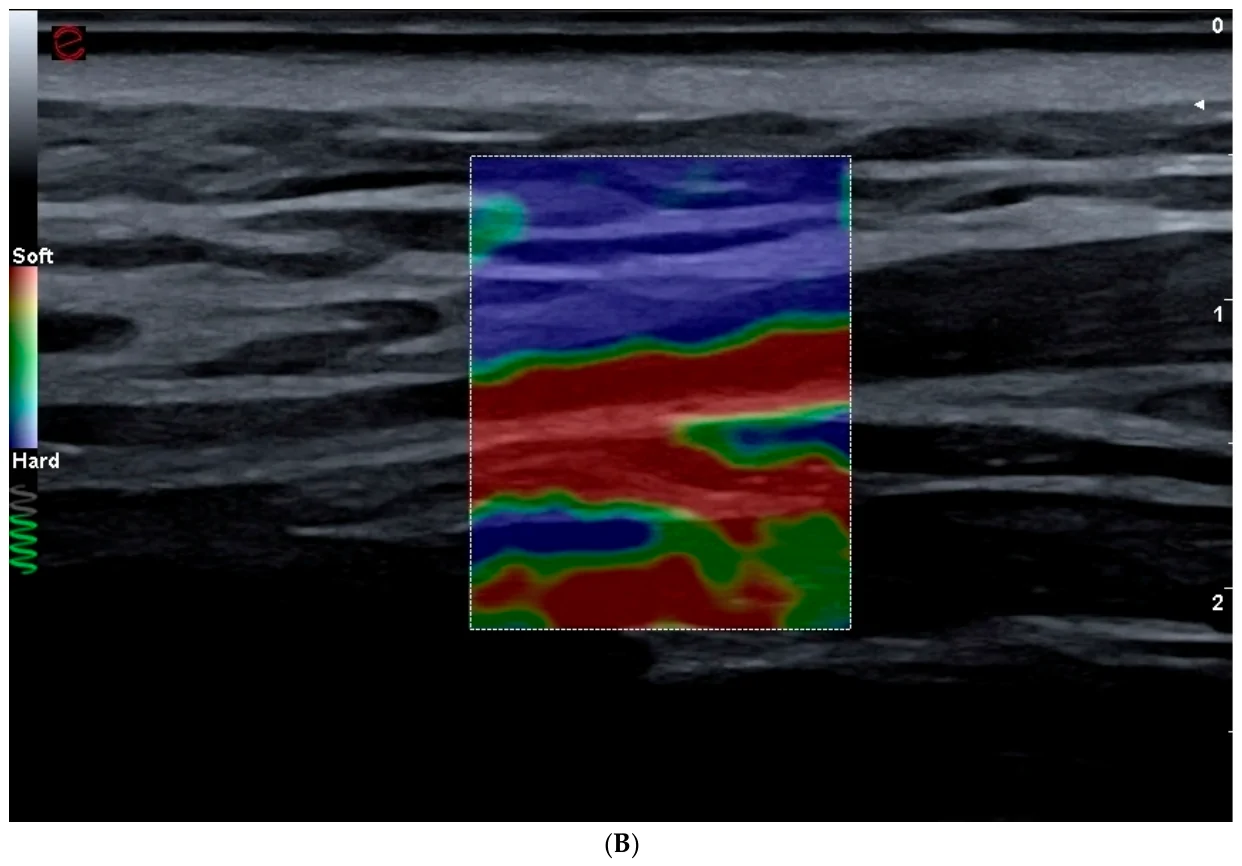
Ultrasound and elastographic evaluation of the cervicodorsal musculature (trapezius muscle): (**A**) B-mode ultrasonography. The region of interest (ROI) delineated by the dotted box presents optimal technical positioning for elastography. It is located strictly within the hypoechoic muscle tissue, excluding the superior and inferior fascial margins. This selectivity avoids contamination of the sample with connective tissue, ensuring that the shear-wave propagation accurately reflects the exclusive stiffness of the muscle belly. (**B**) Study with superimposed Shear-Wave Elastography (SWE) module. At the intramuscular level, a homogeneous color pattern of cold tones (blue, “Soft”) is observed, indicative of low baseline viscoelastic stiffness of the trapezius fibers and good acoustic quality in plane wave transmission. In this specific view, the vertical amplitude of the ROI incorporates the interface of the deep intermuscular fascia at its inferior margin. This fascia is congruently coded in warm tones (reds and greens, “Hard”) given the higher collagen density of the connective tissue, which must be taken into account to avoid an upward bias in the overall quantification of stiffness.

Myofascial pain syndrome is one of the most common chronic musculoskeletal disorders worldwide, with an estimated prevalence of 10–20% in the general population. Clinically, it is characterized by focal or regional pain, taut muscle bands, and trigger points whose palpation elicits or reproduces referred pain [58]. An ultrasound and shear-wave elastography evaluation of the cervicodorsal musculature (trapezius muscle) is shown in Figure 2A,B.

SWE has been used to differentiate active and latent trigger points, although its clinical usefulness for this purpose remains unclear. One study using SWE found no differences in stiffness between active and latent trigger points, including when shear-wave propagation velocity was analyzed [32].

Another study used real-time SWE to investigate differences in the viscoelastic properties of the trapezius muscle between healthy individuals and patients with myofascial pain syndrome. The study included 31 patients with trapezius involvement and 31 healthy volunteers. In patients with myofascial pain syndrome, both the symptomatic trapezius and the unaffected contralateral trapezius showed greater stiffness than those of healthy controls [33].

SWE has also been used to objectively assess the therapeutic response to different treatment techniques in myofascial pain syndrome. One study evaluated muscle stiffness following dry needling and found that resting stiffness of the erector spinae muscles was significantly lower in participants who underwent dry needling than in those who received sham dry needling. However, this difference was observed only at the one-week follow-up and was detected in the erector spinae muscles but not in the lumbar multifidus muscle [34].

Another study used SWE to evaluate the effectiveness of radial extracorporeal shock-wave therapy in upper trapezius myofascial trigger points that had failed to respond to other treatments. The study included 41 patients with a total of 70 active trigger points. In addition to reductions in pain and improvements in cervical function, a decrease in shear modulus was observed in the affected regions. SWE was therefore useful for objectively documenting the therapeutic response [35].

## 9. Plantar Fasciitis

Chronic plantar fasciitis is a degenerative disorder affecting the plantar fascia of the foot as a consequence of repetitive mechanical stress. Its incidence is highest between 40 and 60 years of age, and it is estimated that approximately 1 in 10 individuals in the general population will experience chronic plantar fasciitis at least once during their lifetime [59].

A meta-analysis evaluated the usefulness of SWE for detecting plantar fasciitis. Five studies were included, comprising a total of 158 affected and 134 healthy plantar fasciae. The results showed that pathological plantar fasciae had lower shear-wave propagation velocities and were softer than normal fasciae, indicating that SWE may be useful for distinguishing affected from healthy plantar fascia [36].

A comparative observational study included patients older than 18 years with unilateral heel pain who underwent SWE assessment. No significant differences in plantar fascia thickness were found between the symptomatic and contralateral sides. However, shear-wave velocity was lower on the affected side, reflecting reduced tissue stiffness. These findings suggest that elastography may complement B-mode ultrasonography and facilitate the early detection of plantar fasciitis even when fascial thickness remains normal. SWE demonstrated good reliability in this study under standardized acquisition conditions. However, SWE measurements remain sensitive to several technical and procedural factors, including probe pressure and orientation, ROI placement, acquisition depth, and patient positioning. Despite these limitations, SWE may be useful for monitoring treatment response and for examining whether changes in tissue mechanical properties are associated with changes in pain and function [37].

SWE has also been investigated in the differential diagnosis of plantar fasciitis. One study evaluated the diagnostic usefulness of conventional ultrasonography and SWE for identifying different causes of heel pain. The authors concluded that both techniques can effectively assess this clinical presentation, although conventional ultrasonography should be considered the initial imaging modality of choice, with elastography serving as a complementary method to improve diagnostic accuracy. Furthermore, the addition of SWE facilitated the detection of early plantar fasciitis, and its findings were closely associated with the patients’ clinical course [38].

Another observational case–control study assessed the ability of SWE to detect plantar fasciitis at an early stage. Fifty feet from 33 patients with early-stage disease and 96 asymptomatic feet from 48 healthy volunteers were evaluated. Elasticity parameters measured at the site of greatest plantar fascia thickness showed a positive correlation with pain intensity [39].

SWE has also been used to assess treatment response. One study evaluated the effectiveness of ultrasound-guided corticosteroid injections in patients with plantar fasciitis using SWE. The study included 66 patients aged 27–67 years. Following treatment, plantar fascia stiffness increased significantly, and the elastographic changes were closely associated with clinical improvement [40].

## 10. Tendinopathies

The term tendinopathy encompasses a heterogeneous group of tendon disorders that, although sharing certain clinical and structural features, may have different underlying causes. Tendinopathy is a complex condition involving multiple contributing factors. Epidemiological data from different countries indicate that its prevalence has steadily increased over recent decades among both professional and recreational athletes, as well as among individuals of all ages. This increase may be explained, at least in part, by population aging and the maintenance of higher levels of physical activity at older ages [60].

An experimental ex vivo study used SWE to investigate the effects of structural damage in an animal model of tendinopathy. Sixteen porcine flexor tendons were injected with 0.05 mL of a 1.5% collagenase solution to induce a focal lesion without superficial tearing, while another 16 tendons received saline solution as controls. Collagenase-induced damage resulted in decreased tissue elasticity as measured by SWE. These findings suggest that this technique may be useful for estimating the ultimate mechanical strength of tendons affected by tendinopathy [44].

Although SWE enables non-invasive quantification of tendon elasticity, it has remained uncertain whether tendinopathy-related abnormalities also involve more proximal structures. In a study of 20 patients with chronic Achilles tendinopathy, both limbs were compared using conventional ultrasonography and SWE. A reduction in shear-wave velocity was detected only in the free portion of the tendon, suggesting that alterations in elasticity associated with Achilles tendinopathy are localized to this region and do not extend to the proximal passive tendon structures [45].

Several studies have investigated the role of SWE in different tendinopathies. A systematic review and meta-analysis included 16 studies comprising 676 pathological tendons—188 Achilles, 142 patellar, 96 supraspinatus, and 250 at various anatomical sites—and 723 comparator tendons, of which 484 were from healthy participants and 239 were contralateral tendons. The results suggest that pathological tendons may exhibit lower shear-wave propagation velocities than control tendons, although the certainty of this evidence is very low [16].

**Figure 3:**
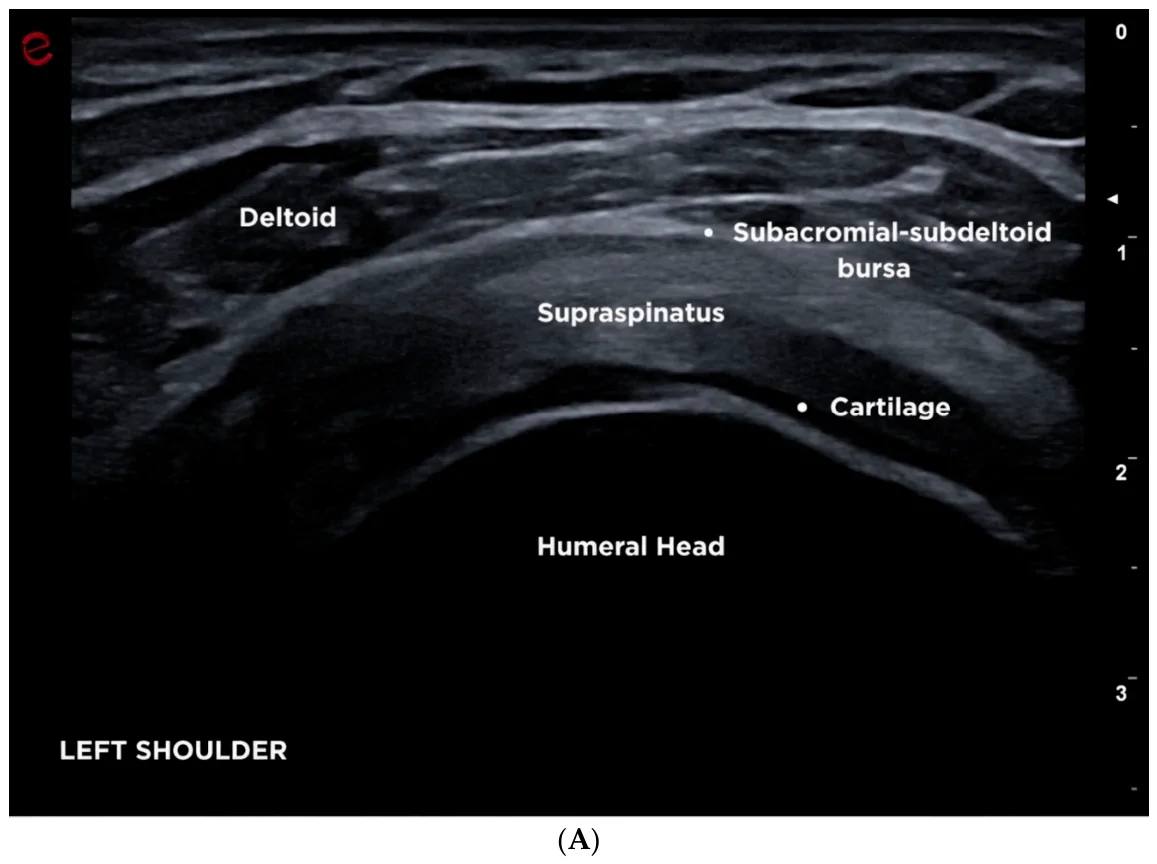

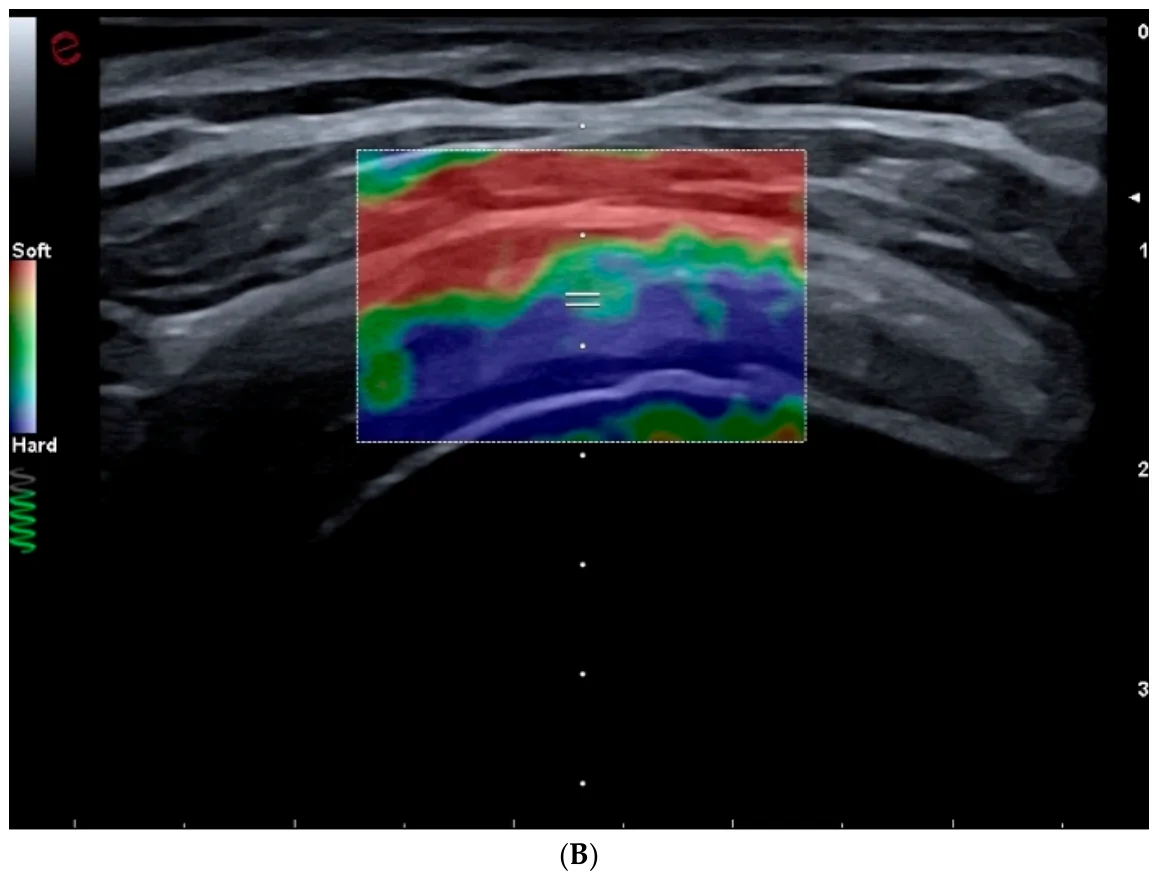
Ultrasound and shear-wave elastography evaluation of the supraspinatus tendon in the short axis (transverse): (**A**) B-mode ultrasonography. Transverse view of the supraspinatus tendon showing its normal convex morphology over the humeral head. (**B**) Evaluation using the Shear-wave elastography module. From superficial to deep, the following tissue biomechanics are interpreted within the region of interest (ROI): Deltoid muscle: predominantly color-coded red. According to the colorimetric scale, this corresponds to low-stiffness tissue (“Soft”), consistent with the normal compressibility of the muscle belly; subacromial-subdeltoid bursa: transitional interface between the deltoid and the tendon, visible in intermediate tones (greens and yellows); supraspinatus tendon: coded almost entirely in dark blue (“Hard” or stiff). This pattern reflects the high collagen density and mechanical resistance typical of a healthy and structurally intact tendon and humeral head cortex marking the deep acoustic boundary. The color pattern suggests preserved tissue biomechanics. In the elastographic evaluation of fibrillar structures, the ultrasound beam is maintained strictly perpendicular to the tendon axis to prevent anisotropy artifacts from introducing false readings of softening (false positives for tendinopathy).

Three systematic reviews have evaluated SWE in rotator cuff disorders. One systematic review of 42 studies analyzed the available evidence regarding the reliability and validity of SWE measurements in rotator cuff muscles. Overall, these measurements showed moderate reliability. However, findings regarding convergent and discriminant validity, as well as responsiveness to changes over time, were heterogeneous. These findings highlight the need to standardize and refine measurement procedures [41]. An ultrasound and shear-wave elastography evaluation of the supraspinatus tendon in the short axis is shown in Figure 3A,B.

Another systematic review investigated the application of SWE for the assessment of rotator cuff tears and included 16 studies with a total of 520 pre- and postoperative patients. Torn supraspinatus tendons showed lower shear-wave propagation velocities than healthy tendons, whereas tendons with tendinopathic changes showed a reduced elastic modulus. Shear-wave velocity also decreased with increasing fatty infiltration and muscle atrophy. One month after surgery, patients who subsequently developed a retear showed higher muscle shear-wave velocities than those in whom the tendon healed successfully [42].

A further systematic review evaluated the usefulness of SWE during the perioperative period in patients undergoing rotator cuff repair. Eleven studies including a total of 355 patients were analyzed. Failure of supraspinatus muscle elasticity to recover was reported as a factor associated with an increased risk of tendon retear. In contrast, preoperative supraspinatus tendon stiffness was not associated with postoperative structural integrity or functional outcomes [43].

The role of SWE has also been investigated in other tendinopathies. One study assessed the ability of SWE to diagnose medial epicondylitis and compared its performance with conventional ultrasonography and strain elastography. Sixty-one elbows from 54 patients were evaluated. The results showed that SWE provides measurements of both tissue stiffness and shear-wave propagation velocity and that these parameters are relevant for diagnosing this tendinopathy [46].

The reliability of SWE in other tendinopathies, such as patellar tendinopathy, remains less well established. One SWE study included 13 patients with patellar tendinopathy, 9 with a history of anterior cruciate ligament reconstruction using a bone–patellar tendon–bone autograft, and 13 participants without previous tendon injury. The results indicated that SWE can provide reliable measurements for quantifying and longitudinally monitoring the viscoelastic properties of the patellar tendon [47].

SWE has also been used as a complementary diagnostic tool in De Quervain tenosynovitis. A prospective SWE study included 19 patients with unilateral clinical involvement and found that SWE measurements were able to differentiate wrists with De Quervain tenosynovitis from unaffected wrists [48].

## 11. Pain Associated with Peripheral Neuropathies

Peripheral neuropathy is characterized by damage to the peripheral nerves and affects approximately 1% of the global adult population. Numerous causes have been described, with highly variable clinical manifestations, some of which can be severely disabling. In Western countries, diabetes is the most common cause. Furthermore, pain may persist despite medical treatment [61].

A meta-analysis evaluated the diagnostic performance of tibial nerve stiffness measured using SWE for detecting diabetic peripheral neuropathy. Six studies were included, comprising 170 patients with diabetic neuropathy, 28 clinically defined cases, 168 patients with diabetes without neuropathy, and 154 healthy participants. The results showed that SWE has good diagnostic accuracy and may be used as a complementary, non-invasive, and clinically useful tool for the assessment of these patients [49].

SWE has been more extensively investigated in entrapment neuropathies. Carpal tunnel syndrome is the most common peripheral entrapment neuropathy and results from sustained compression of the median nerve as it passes through the carpal tunnel. This condition can substantially impair quality of life and work performance, particularly in individuals who perform repetitive wrist movements, and has a higher incidence among women aged 40–60 years. Its most common manifestations include nocturnal paresthesia, progressive sensory loss, and weakness of the thenar muscles, all of which may impair functional use of the hand in daily activities. Diagnosis is based on clinical assessment, provocative maneuvers, and electrophysiological studies, although imaging techniques, particularly conventional ultrasonography and SWE, have gained increasing importance [62].

One study investigated the combined usefulness of B-mode ultrasonography and SWE for determining the severity of carpal tunnel syndrome, using electrodiagnostic studies as the reference standard. The authors reported that elasticity values were significantly higher in moderate or severe cases than in mild cases or individuals without abnormalities, allowing discrimination between these groups [51].

Another study investigated the clinical usefulness of SWE assessment of the transverse carpal ligament in carpal tunnel syndrome and its potential role in guiding subsequent treatment selection. A total of 120 wrists from 60 participants, equally distributed between patient and control groups, were examined. Measurement of transverse carpal ligament stiffness allowed mild cases to be distinguished from those with more advanced disease [52].

A prospective study involving 86 wrists from 47 patients assessed the median nerve using SWE in carpal tunnel syndrome. The authors reported that median nerve shear-wave velocity increased at the carpal tunnel inlet and correlated with neurophysiological severity in a manner similar to nerve enlargement at the same location as measured using conventional ultrasonography [50].

In addition to assessment of the median nerve, SWE has been used to evaluate carpal tunnel syndrome by measuring the stiffness of the abductor pollicis brevis muscle and comparing these findings with electrophysiological studies. Ninety-seven hands were evaluated. Shear-wave velocity in this muscle increased in parallel with the neurophysiological severity of the syndrome and was also higher in the presence of axonal damage, although its diagnostic accuracy was insufficient to identify muscle denervation clinically [53].

SWE has also been used to objectively assess outcomes following decompression surgery. One study compared 50 wrists from 25 healthy volunteers with 54 wrists from 30 surgically treated patients and observed significant changes in both the cross-sectional area and stiffness of the median nerve at the carpal tunnel inlet. Furthermore, both parameters showed a positive correlation with the electrophysiological severity of the disease [54].

SWE has also been investigated for monitoring response to non-surgical treatment. A prospective study including 47 wrists from 41 patients with mild-to-moderate idiopathic carpal tunnel syndrome evaluated the median nerve before and six weeks after ultrasound-guided local corticosteroid injection. Following treatment, median nerve cross-sectional area, shear-wave velocity, and Young’s modulus decreased significantly, accompanied by significant improvements in pain, symptom severity, and functional scores. These findings suggest that SWE may complement clinical outcomes when assessing short-term response to local corticosteroid injection [63].

## 12. Limitations of the Evidence

The current evidence regarding the use of SWE in musculoskeletal pain has several limitations that should be considered.

First, despite the recent expansion of this technique in musculoskeletal disorders, there remains a shortage of studies with high methodological quality, including meta-analyses and systematic reviews, on which to base and support its broader clinical implementation. For example, in tendinopathies, although pathological tendons have been suggested to exhibit reduced shear-wave velocity, the certainty of this evidence remains low [16]. Similarly, although SWE is a potentially valuable technique for assessing the rotator cuff muscles, substantial heterogeneity exists in the literature regarding its validity and its ability to detect changes in tissue properties [41].

In addition to limitations related to study design, SWE itself has several intrinsic technical and physical limitations. SWE assessment is influenced by anatomical factors, the depth of the structures being examined, the size of the ROI, and operator-dependent variables such as the amount of pressure applied, the insonation angle, and probe orientation relative to the muscle fibers [13,14,15].

Musculoskeletal tissues are intrinsically anisotropic and viscoelastic, and their mechanical properties are position-dependent [64]. Because of this anisotropy, the probe must be carefully aligned with the orientation of the tissue fibers, as even small variations can significantly alter the calculated shear modulus [65]. SWE estimates have also been reported to be highly dependent on patient positioning, and considerable variability exists among the algorithms used by different ultrasound system manufacturers [64].

Finally, the vast majority of currently available SWE methods are limited to two-dimensional [2D] imaging. This restriction hampers volumetric analysis of complex anatomical structures subjected to different mechanical loads and may introduce measurement bias when attempting to assess the actual biomechanics of an entire limb or joint [66].

## 13. Future Directions

To consolidate the role of SWE in clinical practice for musculoskeletal disorders, future research should focus primarily on methodological standardization [41].

It is essential to establish tissue-specific measurement protocols, standardize reporting procedures, achieve technical harmonization across ultrasound systems from different manufacturers, and longitudinally validate SWE findings against clinically meaningful outcomes [64].

Similarly, high-quality prospective studies and clinical trials with large sample sizes are required to establish databases of healthy populations, generate reliable reference values, and define accurate cutoff points that may facilitate the early identification of pathological abnormalities.

From a technological and functional perspective, one of the most promising developments is the transition toward three-dimensional [3D] elastography, which could enable volumetric spatial assessments under compressive loading and facilitate the development of computational biomechanical models [66]. Furthermore, the ability of SWE to characterize active and passive mechanics in vivo—including force distribution and the joint-angle-dependent stress–strain behavior of individual muscles—may extend its role beyond that of a non-invasive diagnostic tool toward that of an instrument for functional biomechanical assessment [67].

Beyond the clinical conditions discussed in this review, SWE may also have potential applications in other musculoskeletal structures. For example, a recent study comparing 40 patients with type 2 diabetes mellitus and 40 healthy controls demonstrated significantly increased meniscal stiffness in patients with diabetes, despite the absence of significant differences in meniscal thickness measured using conventional ultrasonography [68]. These findings suggest that SWE may detect alterations in tissue mechanical properties that are not reflected by conventional morphological measurements.

Finally, the prognostic value of SWE warrants further investigation. This includes its potential usefulness for predicting complications or postoperative risks, such as rotator cuff retears [43], and for objectively assessing responses to therapeutic interventions such as injections and extracorporeal shock-wave therapy [35,40].

Overall, the clinical use of SWE should not be regarded as a standalone diagnostic modality but rather as a complementary technique that should be integrated with clinical assessment, neurophysiological evaluation, conventional ultrasonography, and Doppler imaging to maximize diagnostic accuracy in complex musculoskeletal disorders [23,38].

## 14. Conclusions

SWE is an emerging ultrasound-based imaging technique that provides quantitative information on the mechanical properties of musculoskeletal tissues in vivo and may complement the morphological and vascular information obtained with conventional B-mode and Doppler ultrasonography. Current evidence suggests potential clinical applications across a broad range of musculoskeletal disorders, including tendinopathies, plantar fasciitis, mechanical neck and low back pain, myofascial pain, and peripheral neuropathies. Beyond its potential diagnostic value, SWE may also contribute to the objective assessment of disease severity, functional impairment, and tissue changes associated with therapeutic interventions.

Importantly, there is no universal direction of pathological change in SWE measurements. Increased stiffness may characterize some muscle and peripheral nerve disorders, whereas reduced stiffness or shear-wave velocity may be observed in tendinopathy and plantar fasciopathy. Therefore, higher or lower SWE values should not be interpreted independently as markers of pain intensity or disease severity. SWE assesses tissue mechanical properties rather than pain itself, and its findings must always be interpreted within the biological and clinical context of the tissue examined.

However, the clinical interpretation of SWE findings remains challenging. Tissue stiffness is influenced not only by pathological changes but also by anatomical site, tissue anisotropy, muscle contraction, mechanical loading, patient positioning, acquisition depth, probe pressure and orientation, and the characteristics of the ultrasound system used. Moreover, substantial methodological heterogeneity exists among studies, and for several musculoskeletal conditions the available evidence is still derived mainly from observational studies with relatively small sample sizes. Consequently, SWE should not currently be considered a standalone diagnostic modality, but rather a complementary tool whose findings should be interpreted together with clinical examination, conventional ultrasonography, Doppler imaging, and, when appropriate, neurophysiological assessment.

Further development of SWE in musculoskeletal medicine will require standardized tissue-specific acquisition protocols, harmonization of measurements across manufacturers, reliable reference values and clinically meaningful cutoff points, and validation in large prospective studies and high-quality clinical trials. Future advances, including three-dimensional elastography and the assessment of active and passive tissue biomechanics, may further expand its applications. In particular, establishing the prognostic value of SWE and its ability to objectively monitor treatment response could facilitate its transition from a predominantly research-oriented imaging technique to a clinically relevant tool for diagnosis, functional assessment, prognosis, and longitudinal follow-up of musculoskeletal disorders.

## Figures and Tables

**Table 1:** Summary of shear-wave elastography (SWE) findings and potential clinical applications by musculoskeletal pain condition.

Condition	Tissue(s) Assessed	Population	Stiffness vs. Healthy Tissue	Main Findings	Potential Clinical Applications	Studies Reviewed (References)
**Mechanical neck pain**	Anterior scalene, upper trapezius	Patients with mechanical or chronic neck pain, including office workers; asymptomatic individuals	Higher (muscle)	Stiffness associated with pain severity, disability and fear-avoidance beliefs; limited ability to discriminate patients from asymptomatic individuals	Complementary assessment; not recommended as a standalone technique	Five studies: four comparing patients with asymptomatic individuals or healthy controls and one correlation study [22,23,24,25,26]
**Mechanical low back pain**	Lumbar muscles (quadratus lumborum, erector spinae, multifidus, psoas major), thoracolumbar fascia, rectus abdominis	Patients with nonspecific low back pain; elite athletes with and without chronic low back pain; pregnant women; healthy individuals	Higher (muscle and fascia)	Differences more pronounced at L4–L5; stiffness possibly associated with pain intensity and disability; excellent intra-rater reproducibility for the quadratus lumborum	Classification as acute, subacute or chronic; prediction of postpartum low back pain	Five studies: one reliability study [27], one cross-sectional study [28], one comparison of athletes with and without pain [29], one classification study [30] and one risk prediction model [31]
**Myofascial pain**	Upper trapezius, erector spinae	Patients with myofascial pain syndrome or active trigger points; healthy volunteers	Higher (muscle)	Higher stiffness also in the unaffected contralateral trapezius; no differences between active and latent trigger points; lower stiffness after dry needling and shockwave therapy	Monitoring of treatment response (dry needling, radial extracorporeal shockwave therapy)	Four studies: one comparing active and latent trigger points [32], one comparing patients with healthy volunteers [33], one sham-controlled study of dry needling [34] and one study of shockwave therapy [35]
**Plantar fasciitis**	Plantar fascia	Patients with heel pain or plantar fasciitis, including early-stage disease; healthy volunteers	Lower (fascia)	Lower velocity even when fascial thickness is normal; stiffness increases with clinical improvement after corticosteroid injection	Early and differential diagnosis, complementary to B-mode ultrasound; monitoring of response to corticosteroid injection	One meta-analysis [36], one comparative observational study [37], one diagnostic study of heel pain [38], one observational case–control study [39] and one study of corticosteroid injection [40]
**Tendinopathies**	Achilles tendon; patellar tendon; rotator cuff tendons and muscles; elbow (medial epicondylitis); wrist (De Quervain tenosynovitis)	Patients with Achilles or patellar tendinopathy, rotator cuff disorders, medial epicondylitis or De Quervain tenosynovitis; healthy participants; porcine flexor tendons (ex vivo)	Lower (tendon)	Lower velocity in pathological tendons, with very low certainty of evidence; in Achilles tendinopathy, changes limited to the free portion of the tendon; failure of muscle elasticity to recover after rotator cuff repair associated with retear	Complementary diagnosis; prediction of retear and perioperative monitoring after rotator cuff repair	One systematic review and meta-analysis [16], three systematic reviews [41,42,43], one experimental ex vivo study [44], one study comparing symptomatic and contralateral tendons [45], one diagnostic study [46], one reliability study [47] and one prospective study [48]
**Peripheral neuropathies**	Tibial nerve (diabetic neuropathy); median nerve, transverse carpal ligament and abductor pollicis brevis (carpal tunnel syndrome)	Patients with diabetes, with or without neuropathy, or with carpal tunnel syndrome; healthy participants	Higher (nerve and muscle)	Good diagnostic accuracy for diabetic peripheral neuropathy; in carpal tunnel syndrome, stiffness associated with disease severity	Complementary diagnosis of diabetic neuropathy; severity grading and treatment selection in carpal tunnel syndrome; assessment after decompression surgery	One meta-analysis [49], one prospective study [50] and four studies comparing SWE with electrophysiological severity or healthy controls [51,52,53,54]

Stiffness refers to shear-wave velocity or elastic modulus in affected tissue compared with healthy or contralateral tissue. The direction of change is tissue- and condition-specific; higher or lower SWE values should not be interpreted as a generic marker of pain or disease severity across tissues.

## Data Availability

No new data were created or analyzed in this study. Data sharing is not applicable to this article.
